# Antibiotic prescription for under-fives with common cold or upper respiratory tract infection in Savannakhet Province, Lao PDR

**DOI:** 10.1186/s41182-019-0143-z

**Published:** 2019-02-28

**Authors:** Bounxou Keohavong, Manithong Vonglokham, Bounfeng Phoummalaysith, Viengsakhone Louangpradith, Souphalak Inthaphatha, Tetsuyoshi Kariya, Yu Mon Saw, Eiko Yamamoto, Nobuyuki Hamajima

**Affiliations:** 1 Department of Healthcare Administration, Nagoya University Graduate School of Medicine, 65 Tsurumai-Cho, Showa-Ku, Nagoya, 466-8550 Japan; 2 Food and Drug Department, Ministry of Health, Simuang road, Vientiane Capital, Lao People’s Democratic Republic; 3 Lao Tropical and Public Health Institute, Ministry of Health, Vientiane Capital, Lao People’s Democratic Republic; 4 National Health Insurance Bureau, Ministry of Health, Vientiane Capital, Lao People’s Democratic Republic; 5 Mittaphab Hospital, Phonsavang V, Chanthabouly D, Vientiane Capital, Lao People’s Democratic Republic

**Keywords:** Prescribing practice, Antibiotics, Common cold, Upper respiratory tract infections, Lao PDR

## Abstract

**Background:**

The irrational use of antibiotics has been identified as a major problem in healthcare, and it can lead to antimicrobial resistance, treatment failure, and increased healthcare costs. Although many studies worldwide have focused on the irrational use of drugs, reports on prescription practice in Lao PDR remained limited. This study aimed to examine the patterns of antibiotic prescription for under-fives with common cold or upper respiratory tract infection (URTI) at pediatric outpatient divisions.

**Methods:**

One provincial hospital (PH) at Kaisone Phomvihane and four district hospitals (DHs) at Songkhone, Champhone, Atsaphangthong, and Xepon in Savannakhet Province were selected. Healthcare providers at these hospitals were interviewed and medical records of under-fives from 2016 were examined.

**Results:**

Of the 54 healthcare providers interviewed, 85.2% had seen the standard treatment guideline, 77.8% adhered to this guideline, and 90.7% knew about antimicrobial resistance, while 18.5% participated in antimicrobial resistance activities. Medical records of 576 outpatients (311 boys and 265 girls) with common cold or URTI were examined, 154 at the PH and 422 at the DHs. Although antibiotics prescription proportions were similar between facilities at both levels (68.8% and 70.9% at the PH and DHs, respectively), antibiotics were exclusively prescribed for URTIs (96.4%), not for common cold (4.9%). First-line antibiotics recommended by WHO Model List of Essential Medicines for Children the 6th List were prescribed for 81.5% of patients; mainly, beta-lactam antibiotics were prescribed (87.2% of prescribed antibiotics). There were no cases in which two or more antibiotics were prescribed. The correct dose according to the National STG was 31.9% as a whole. The difference in the correct dose between the PH (52.8%) and the DHs (24.4%) was significant (*p* < 0.001).

**Conclusions:**

This study demonstrated the patterns of antibiotic prescription for under-fives with common cold or URTI among healthcare providers from two different levels of facilities. Although an appropriate number of generic first-line antibiotics in the essential drug list were prescribed, the dosage and duration of antibiotic use were not appropriate. In order to further improve antibiotic prescription practices, regulation by the government is necessary; this could also decrease antimicrobial resistance and improve treatment outcomes.

## Introduction

Mortality from infectious diseases is a major health issue, especially among children. The appropriate use of antibiotics is critical for pediatric medical care. Upper respiratory tract infections (URTIs) are commonly observed among children under 5 years of age (U5) for whom antibiotics have been frequently prescribed. Self-medication with antibiotics is also commonly observed in many developing countries, and unnecessary medication and inappropriate antibiotic use can lead to antimicrobial resistance, treatment failure, and increased healthcare costs [1–4]. Thus, healthcare providers need to pay close attention to inappropriate antibiotic prescription [5, 6], and policy makers should develop efficient strategies to reduce such inappropriate prescriptions [7].

Several studies on community antibiotic use have been performed in high-income countries [8, 9]. Among the developing countries, antibiotic overprescription has been reported in Pakistan and India, as well as in several African countries [10, 11]. The overuse of drugs, linked to inappropriate prescription practices, may increase health cost budgets in countries aiming for universal health coverage.

Studies addressing this issue in Lao People’s Democratic Republic (Lao PDR) are limited. Available studies have revealed the prevalence of inappropriate antibiotic use practices in private pharmacies in Lao PDR [12–14]. The irrational use of medicines is frequently observed in developing countries. In Lao PDR, unnecessary antibiotic prescriptions were found to be frequent; the 2016 annual report on the treatment of tracer diseases in four northern provinces revealed that 70% of U5 with usual diarrhea received antibiotics on visiting an outpatient division (OPD). A survey of prescribers and prescriptions in Lao PDR revealed the considerable use of polypharmacy and overuse of antibiotics [13].

Lao PDR showed substantial progress in decreasing the mortality rate among U5, achieving Millennium Development Goal 4 in 2015 [15]. The U5 mortality declined from 163 per 1000 in 1990 to 70 per 1000 in 2012 [16]. During this period, under the National Drug Policy Programme, the Ministry of Health (MoH), Lao PDR recommended all provincial and district hospitals to establish their own drug and therapeutics committee (DTC). In addition, indicators to evaluate DTC performance and Standard Treatment Guideline (STG) use were developed for measuring the quality of treatment, rational use of drugs (RUD) during prescription, and better hospital management [17]. This study aimed to examine knowledge and practices, as well as the practices related to antibiotic prescription for U5 with common cold or URTI in pediatric OPDs in Savannakhet Province.

## Materials and methods

### Participants

Savannakhet is the largest (21,774 km^2^) and most populous province of Lao PDR, with 1,001,000 inhabitants (512,000 males and 489,000 females) in 2017. Savannakhet has 15 districts. Among those, one district (Kaisone Phomvihane) has a tertiary provincial hospital (PH) and four districts (Songkhone, Champhone, Atsaphangthong, and Xepon) have one secondary district hospitals (DHs) each. The remaining ten districts have no tertiary/secondary hospitals [18].

Healthcare providers were selected through convenient sampling among medical staff at pediatric OPDs providing healthcare for U5 at the PH and four DHs. They were aged 22 to 57 years, and their experience as healthcare providers ranged from 2 months to 36 years. Interviews were conducted by two researchers. U5 outpatients diagnosed with common cold or URTI were sampled from the list of outpatients who visited the hospitals during the first week (five consecutive days, Monday to Friday) of each month in 2016 (in total 60 days). Common cold was defined as symptoms of inflammation in the nose and throat mucous membrane with body temperature exceeding 37 °C, but without a sore throat. URTI including pharyngitis, otitis media, and tonsillitis was defined as the presence of a sore throat, difficulty in swallowing, red throat, white particles in the throat, and/or earaches, with a body temperature exceeding 37 °C, and white blood cell counts of more than 9000 cells/μl.

### Questionnaire

The questionnaire administered to healthcare providers consisted of four main items: (1) characteristics of the interviewee, (2) knowledge about prescription, (3) prescription practice and expectation on prescription, and (4) belief in antibiotic prescription practice. The questionnaire was pretested at Vientiane Provincial Hospital. The above information was collected with a face-to-face interview using the questionnaire during 25–30 min per interviewee.

Information of U5 outpatients was collected using a data form from medical charts. This included information on (1) sex, (2) age, (3) health insurance scheme, (4) diagnosis, (5) number of prescribed drugs, (6) number of prescribed antibiotics, (7) drug name, (8) examination and tests before diagnosis, and (9) rationale of prescription practice based on the STG recommendations.

### Standard treatment guideline in Lao PDR

The first version of the National STG was developed in 1998, with funding from the Swedish International Development Agency. The Healthcare Department in cooperation with the Food and Drug Department of MoH was responsible for developing the National STG. Since the healthcare providers in Lao PDR were graduates from medical schools in domestic and foreign countries, their knowledge and practice were based on different standards. The first version of the National STG was developed for seven selected leading causes of morbidity and mortality in Lao PDR (malaria, diarrhea, upper and lower respiratory tract infection, tuberculosis, dengue, leprosy, and parasite infections). It was revised to address 16 diseases including common cold, with the latest revision (2012) addressing 255 diseases [19]. The National STG is distributed among all levels of health facilities in Lao PDR. It has been used by healthcare providers as a reference for treatment and prescription. The recent STG for URTI recommends a 10-day regime consisting of (1) penicillin V 50,000–100,000 UI/kg/day, four times daily, (2) erythromycin 40 mg/kg/day, four times daily in cases of allergy to penicillin V, or (3) amoxicillin 30-50 mg/kg/day, three times daily, as well as antipyretic (paracetamol) medication and recommendations for sufficient water and food intake and relaxation.

### Statistical analysis

CSPro version 7.1 was used for data entry, and IBM Statistical Package for Social Sciences (SPSS) version 20 was used for statistical analysis. Categorical data were examined using a chi-square test. The 95% confidence interval (CI) of the percentage was calculated using the binomial distribution. An unconditional logistic model was applied to estimate odds ratios (ORs) and their 95% CIs. A *p* value less than 0.05 was considered statistically significant.

## Results

### Usage of the guidelines

In total, 54 healthcare providers (20 males and 34 females) at five health facilities (6 at one PH and 48 at four DHs) were interviewed. These individuals were the staff involved in medical check-ups at outpatient wards; 22 (40.7%) medical doctors, 4 (7.4%) pediatricians, 11 (20.4%) general practitioner, and 17 (31.5%) primary healthcare staff (Table 1). A bachelor’s degree was the most common qualification (35.2%) among the interviewees, followed by medical assistant (16.7%). Further, 11.1% were medical specialist level 1, healthcare providers with an additional specialization in a certain medical field with 3-year post graduation training. Forty (74.1%) interviewees were permanent employees, while 13 (24.1%) were volunteers. One third of the healthcare providers worked at outpatient wards, and the rest worked in emergency units and inpatient wards. All of them had been providing healthcare services to U5 at public health facilities; half of them had been providing healthcare services to U5 at a private health facility as a part-time job (51.9%).

**Table 1:** Characteristics of healthcare providers interviewed

Characteristics	Male	Female	Total
	*N* (%)	*N* (%)	*N* (%)
Healthcare providers interviewed	20 (100)	34 (100)	54 (100)
Facility			
Provincial hospital	1 (5.0)	5 (14.7)	6 (11.1)
District hospitals	19 (95.0)	29 (85.3)	48 (88.9)
Medical background			
Medical doctors	12 (60.0)	10 (29.4)	22 (40.7)
Pediatricians	1 (5.0)	3 (8.8)	4 (7.4)
General practitioners^a^	1 (5.0)	10 (29.4)	11 (20.4)
Primary healthcare staff ^b^	6 (30.0)	11 (32.4)	17 (31.5)
Professional level			
Master degree	0 (0.0)	1 (2.9)	1 (1.9)
Bachelor	9 (45.0)	10 (29.4)	19 (35.2)
Specialist level 1^c^	3 (15.0)	3 (8.8)	6 (11.1)
Medical assistant	2 (10.0)	7 (20.6)	9 (16.7)
Other	6 (30.0)	13 (38.2)	19 (35.2)
Employee status			
Employed	12 (60.0)	28 (82.4)	40 (74.1)
Work contact	0 (0.0)	1 (2.9)	1 (1.9)
Voluntary staff	8 (40.0)	5 (14.7)	13 (24.1)
Working division			
OPD	4 (20.0)	11 (32.4)	15 (27.8)
IPD	9 (45.0)	9 (26.5)	18 (33.3)
Emergency department	7 (35.0)	14 (41.2)	21 (38.9)
Work experiences/practice			
Heath check-up for U5	20 (100.0)	34 (100.0)	54 (100.0)
Health service outside HF	11 (55.0)	17 (50.0)	28 (51.9)

^a^General medical staff with a bachelor degree graduated from a medical school

^b^Staff graduated from 2-year primary healthcare school

^c^Staff with additional specialization after a 3-year training

*HF* health facility, *OPD* outpatient division, *IPD* inpatient division, *U5* children under 5 years of age

Table 2 shows the knowledge of the healthcare providers according to sex. Most providers had seen the STG for children (98.1%, 95% CI 90.1–99.9%), and applied it for the prescription (96.3%, 95% CI 87.2–99.5%). Further, 87.0% (95% CI 75.1–94.6%) had heard about DTC, only 22.2% were DTC members, and 61.1% (95% CI 46.9–74.1%) had contributed to the DTC’s activities in their own health facilities. Among the 54 healthcare providers, 72.2% (95% CI 58.4–83.5%) had seen the essential drug list (EDL), 92.6% (95% CI 82.1–97.9%) had heard about adverse drug reactions (ADR), and 90.7% (95% CI 79.7–96.9%) had heard about antimicrobial resistance. Moreover, 44.4% (95% CI 30.9–58.6%) had heard about the Antibiotic Awareness Week (AAW), and 18.5% (95% CI 9.2–31.4%) had participated in AAW activities at least once. Approximately seven eighths of them had been trained in medical practice in the last 2 years (85.2%, 95% CI 72.9–93.4%), but less than half had received RUD training (46.3%, 95% CI 32.6–60.4%). The percentage was significantly higher among males (65.0%, 95% CI 40.8–84.6%) than among females (35.3%, 95% CI 19.7–53.5%).

**Table 2:** Knowledge of healthcare providers (20 males and 34 females): those who answered “yes” to each question

Question	Male	Female	Total
	*N* (%)	*N* (%)	*N* (%)
Have ever seen main STG	18 (90.0)	28 (82.4)	46 (85.2)
Have ever seen STG for children	19 (95.0)	34 (100.0)	53 (98.1)
Using main STG in prescribing medicines	15 (75.0)	27 (79.4)	42 (77.8)
Using STG for children in prescribing medicines	20 (100.0)	32 (94.1)	52 (96.3)
Have ever heard about DTC	17 (85.0)	30 (88.2)	47 (87.0)
Member of the DTC	4 (20.0)	8 (23.5)	12 (22.2)
Have ever been contributed to DTC performance	10 (50.0)	23 (67.6)	33 (61.1)
Have ever seen EDL	13 (65.0)	26 (76.5)	39 (72.2)
Have ever heard about ADR	18 (90.0)	32 (94.1)	50 (92.6)
Have contribute to ADR reporting	3 (15.0)	11 (32.4)	14 (25.9)
Have ever heard about AAW	10 (50.0)	14 (41.2)	24 (44.4)
Have been joined AAW activity	4 (20.0)	6 (17.6)	10 (18.5)
Have ever heard about antimicrobial resistant	18 (90.0)	31 (91.2)	49 (90.7)
Have ever been trained on medical practice	18 (90.0)	28 (82.4)	46 (85.2)
Have ever been trained RUD*	13 (65.0)	12 (35.3)	25 (46.3)

*STG* standard treatment guideline, *DTC* drug and therapeutics committee, *EDL* essential drug list, *ADR* adverse drug reaction, *AAW* Antibiotic Awareness Week, *RUD* rational use of drugs,

*p < 0.05 between males and females

Table 3 shows the prescription practices and expectation on prescription among the 54 healthcare providers. In all, 5.6% (95% CI 1.2–15.4%) stated that they performed blood tests for every outpatient. Antibiotics were prescribed by relying on symptoms in 98.1% (95% IC 90.1-99.9%) of cases. Although 92.6% (95% CI 82.1–97.9%) of providers reported having confidence in the prescription, 72.2% (95% CI 58.4–83.5%) had concerns about the quality of antibiotics, 79.6% had concerns about patients following their advice, 85.2% (95% CI 72.9–93.4%) had concerns about self-medication of antibiotics at the next occurrence of similar symptoms, and 90.7% (95% CI 79.7–96.9%) had concerns about the adverse effects of antibiotics and antimicrobial resistance. While almost all healthcare providers believed in treatment guidelines (98.1%, 95% CI 90.1–99.9%), 61.1% (95% CI 46.9–74.1%) strongly agreed with the effectiveness of the regulation, and 64.8% (95% CI 50.6–77.3%) believed in the prioritizing careful examination (Table 4).

**Table 3:** Prescription practices and expectation on prescription among healthcare providers (20 males and 34 females)

	Male	Female	Total
	*N* (%)	*N* (%)	*N* (%)
Laboratory examination			
Blood test for every case	2 (10.0)	1 (2.9)	3 (5.6)
Blood testing for selected cases	17 (85.0)	30 (88.2)	47 (87.0)
Based on symptoms	20 (100.0)	34 (100.0)	54 (100.0)
Based on full examination	16 (80.0)	24 (70.6)	40 (74.1)
In case of an enough time available	15 (75.0)	30 (88.2)	45 (83.3)
Reason for AB prescription			
Base on observed symptoms	20 (100.0)	33 (97.1)	53 (98.1)
Just for prevention	2 (10.0)	0 (0.0)	2 (3.7)
Based on experiences	5 (25.0)	5 (14.7)	10 (18.5)
Expectation from prescribing practice			
Being confident for AB prescription	20 (100.0)	30 (88.2)	50 (92.6)
Have concerns about quality of AB prescribed	17 (85.0)	22 (64.7)	39 (72.2)
Have concerns about compliance to the advice	18 (90.0)	25 (73.5)	43 (79.6)
Have concerns about self-medication of AB	18 (90.0)	28 (82.4)	46 (85.2)
Have concerns about AB resistant	19 (95.0)	30 (88.2)	49 (90.7)
Have concerns about adverse effects and resistance	20 (100.0)	29 (85.3)	49 (90.7)

*AB* antibiotics

**Table 4:** Belief in antibiotic prescription practices among healthcare providers (20 males and 34 females)

Belief	Male	Female	Total
	*N* (%)	*N* (%)	*N* (%)
What is the most important to contribute effective AB prescription			
Following the guidelines	20 (100.0)	33 (97.1)	53 (98.1)
Full examination before prescription	20 (100.0)	32 (94.1)	52 (96.3)
Ethical prescription	19 (95.0)	33 (97.1)	52 (96.3)
Do you agree that AB prescription regulation can change prescribers’ behaviour to better prescription practices			
Strongly agree	12 (60.0)	21 (61.8)	33 (61.1)
Agree	5 (25.0)	8 (23.5)	13 (24.1)
Not sure	3 (15.0)	5 (14.7)	8 (14.8)
The priority message to prescribers on AB prescription			
Careful examination	12 (60.0)	23 (67.6)	35 (64.8)
Avoidance of AB prescription if possible	3 (15.0)	5 (14.7)	8 (14.8)
Full laboratory examine	2 (10.0)	3 (8.8)	5 (9.3)
Practices based on guidelines	3 (15.0)	1 (2.9)	4 (7.4)
Prescription of first-line AB if necessary	0 (0.0)	2 (5.9)	2 (3.7)
The priority message for health policy makers to act on AB prescription practice improvement			
Development of AB prescription regulation	12 (60.0)	16 (47.1)	28 (51.9)
Advocacy to related health staff	3 (15.0)	7 (20.6)	10 (18.5)
Strong promotion of RUD	3 (15.0)	6 (17.6)	9 (16.7)
Awards for good prescribers	1 (5.0)	3 (8.8)	4 (7.4)

*AB* antibiotics, *RUD* rational use of drugs

### Antibiotic prescription based on medical records

Information on antibiotic prescription was collected for 576 children (311 boys and 265 girls) who were diagnosed with common cold or URTI in 2016 at the OPD of Savannakhet PH (154 patients) and four DHs (422 patients). Among U5, the 1-year age group was the largest (25.2%, 95% CI 21.7–28.9%), while the 4-year age group was the smallest (12.3%, 95% CI 9.8–15.3%), as shown in Table 5. Further, 39.4% (95% CI 35.4–43.5%) of the patients were not covered by health insurance. Approximately 70% of the 349 insured patients were covered by community-based health insurance. Moreover, 31.2% (95% CI 24.0–39.1%) and 27.5% (95% CI 23.3–32.0%) were diagnosed with common cold at the PH and the DHs, respectively.

**Table 5:** Characteristics of outpatients under 5 years of age at provincial hospital (PH) and district hospitals (DH)

Characteristics	PH	DH	Total
	*N* (%)	*N* (%)	*N* (%)
Total	154 (100)	422 (100)	576 (100)
Boys	74 (48.1)	237 (56.2)	311 (54.0)
Girls	80 (51.9)	185 (43.8)	265 (46.0)
Age group			
0 year	29 (18.8)	98 (23.2)	127 (22.0)
1 year	31 (20.1)	114 (27.0)	145 (25.2)
2 years	42 (27.3)	93 (22.0)	135 (23.4)
3 years	34 (22.1)	64 (15.2)	98 (17.0)
4 years	18 (11.7)	53 (12.6)	71 (12.3)
Health insurances status			
Covered by health insurance	90 (58.4)	259 (61.4)	349 (60.6)
SASS*	22 (14.3)	24 (5.7)	46 (8.0)
SSO**	31 (20.1)	14 (3.3)	45 (7.8)
CBHI**	37 (24.0)	201 (47.6)	238 (41.3)
HEF*	0 (0.0)	21 (5.0)	21 (3.6)
Not covered by health insurance	64 (41.6)	163 (38.6)	227 (39.4)
Symptomatic of outpatient			
Common cold	48 (31.2)	116 (27.5)	164 (28.5)
Pharyngitis	92 (59.7)	26 (62.3)	355 (61.6)
Otitis media	0 (0.0)	7 (1.7)	7 (1.2)
Tonsilitis/amygdalite	14 (9.1)	36 (8.5)	50 (8.7)

*SASS* state authority for social security, *SSO* social security organization, *CBHI* community-based health insurance, *HEF* health equity fund

*p < 0.01

**p < 0.001 between PH and DH

Table 6 shows the number of drug items prescribed for U5. On average, 2.4 drug items (375 items) were prescribed for 154 outpatients at the PH, and 2.8 drug items (1166 items) were prescribed for 422 outpatients at the DHs. At the PH, essential drugs were prescribed for 83.4% (95% CI 79.3–87.1%) of U5 patients, and 77.6% (95% CI 73.0–81.7%) were prescribed drugs using a generic name (international non-proprietary name; INN). Antibiotics were prescribed for 106 (68.8%, 95% CI 60.9–76.0%) patients, and nobody was prescribed more than one antibiotic. First-line antibiotics (amoxicillin, ampicillin, erythromycin, and penicillin V) were prescribed for 97.2% (95% CI 91.9–99.4%) of patients. Moreover, beta-lactam antibiotics were prescribed for 87.7% (95% CI 79.4–93.3%), while macrolides were prescribed for 9.4% (95% CI 4.6–16.7%). Among the 1166 drugs at the DHs, 91.5% (95% CI 89.9–93.1%) were from EDL, and 81.7% (95% CI 79.4–83.9%) were generic drugs. Antibiotics were prescribed for 299 (70.9%, 95% CI 66.3–75.1%) patients. Even at the DHs, nobody was prescribed more than one antibiotic. First-line antibiotics were prescribed for 97.7% of the U5. The beta-lactams, macrolides, and cephalosporins were prescribed 87.0% (95% CI 82.6–90.6%), 11.0% (95% CI 7.7–15.1%), and 1.7% (95% CI 0.5–3.9%), respectively. Among the first-line antibiotics, 87.7% (95% CI 79.9–93.3%) and 79.3% (95% CI 74.2–83.7%) at the PH and DHs, respectively, were recommended by WHO Model List of Essential Medicines for Children the 6th List. The correct dose based on the National STG was administered to 52.8% (95% CI 42.9–62.6%) at the PH and 24.4% (95% CI 19.7–29.7%) at the DHs, and the appropriate duration was prescribed for 2.2% (95% CI 1.0–4.2%) of U5.

**Table 6:** Drug prescriptions for outpatients under 5 years of age at provincial hospital (PH) and district hospitals (DH)

Variables	PH	DH	Total
	*N* (%)	*N* (%)	*N* (%)
All prescribed drugs	375 (100)	1166 (100)	1541 (100)
EDL drugs	313 (83.4)	1068 (91.5)	1381 (89.6)
INN drugs	291 (77.6)	953 (81.7)	1244 (80.7)
AB medication	106 (100)	299 (100)	405 (100)
First-line antibiotics based on National STG	103 (97.2)	292 (97.7)	395 (97.5)
Drugs other than the first-line antibiotics	3 (2.8)	7 (2.3)	10 (2.5)
More than one antibiotic	0 (0.0)	0 (0.0)	0 (0.0)
Antibiotic family			
Beta-lactams	93 (87.7)	260 (87.0)	353 (87.2)
Cephalosporins	2 (1.9)	5 (1.7)	7 (1.7)
Macrolides	10 (9.4)	33 (11.0)	43 (10.6)
Sulfonamides	1 (0.9)	1 (0.3)	2 (0.5)
AB prescription			
First-line AB based on WHO MLEMC	93 (87.7)	237 (79.3)	330 (81.5)
Correct doses based on the National STG*	56 (52.8)	73 (24.4)	129 (31.9)
Appropriate duration based on the National STG	0 (0.0)	9 (3.0)	9 (2.2)

WHO MLEMC: WHO Model List of Essential Medicines for Children the 6th List (March 2017). First-line antibiotics of the National STG include amoxicillin, ampicillin, erythromycin, and penicillin V. The first-line antibiotics based on the WHO MLEMC includes amoxicillin and penicillin V

*AB* antibiotics, *EDL* essential drug list, *INN* international non–proprietary name, *SD* standard deviation, *STG* standard treatment guideline

*p < 0.001 between PH and DH

Table 7 shows the ORs and 95% CIs of antibiotic prescription for U5 with common cold or URTI. Antibiotics were prescribed for eight (4.9%) out of 164 patients with common cold, 341 (96.0%) out of 355 patients with pharyngitis, and 56 (98.3%) out of 58 patients with other URTI. After adjustment, factors other than diagnosis were not significantly associated with antibiotic prescription.

**Table 7:** Odds ratios (ORs) and 95% confidence intervals (CIs) of antibiotic prescription for outpatients under 5 years of age with common cold or upper respiratory tract infection

Characteristics	AB prescribed	Unadjusted	Adjusted
	*N* (%)	OR (95% CI)	OR (95% CI)
Hospital			
Provincial	106 (68.8)	1 (Reference)	1 (Reference)
District	299 (70.8)	1.10 (0.73–1.64)	0.73 (0.26–2.07)
Sex			
Boys	217 (69.8)	1 (Reference)	1 (Reference)
Girls	188 (70.9)	1.06 (0.74–1.51)	1.04 (0.44–2.46)
Age group			
0 year	79 (62.2)	1 (Reference)	1 (Reference)
1 year	99 (68.3)	1.30 (0.79–2.16)	0.55 (0.15–2.06)
2 years	102 (75.6)	1.88 (1.10–3.19)	0.72 (0.18–2.81)
3 years	74 (75.5)	1.87 (1.04–3.36)	0.40 (0.93–1.71)
4 years	51 (71.8)	1.54 (0.82–2.91)	0.30 (0.06–1.41)
Health insurances status			
Covered by CBHI	180 (73.6)	1 (Reference)	1 (Reference)
Covered by the others	85 (75.8)	1.41 (0.88–2.28)	1.48 (0.42–5.20)
Not covered by health insurance	141 (62.1)	0.53 (0.37–0.76)	1.37 (0.49–3.82)
Diagnosis			
Common cold	8 (4.9)	1 (Reference)	1 (Reference)
Pharyngitis	341 (96.0)	474 (195–1155)	660 (234–1856)
Others	56 (98.3)	1092 (133–8927)	1567 (177–13,876)

*CBHI* community-based health insurance

## Discussion

### Usage of the guideline

This study demonstrated a high usage of the National STG (77.8%), especially for children (96.3%). The guideline was designed to be convenient for healthcare providers. The usage in our study was higher than that in Ghana (41.7%) [20], South Africa (45.1%) [21], and Israel (55.7%) [22]. Most healthcare providers had heard about DTC (87.0%), but only 22.2% were DTC members. Although the EDL had been distributed among all levels of health facilities, the current study found that 72.2% of the interviewees confirmed having seen this list, which was meant to be used as a reference for the procurement and prescription. Most interviewees stated that they had heard about ADR (92.6%) and antimicrobial resistance (90.7%), possibly because Lao PDR became an official member of the WHO Uppsala Monitoring Center in July 2015, due to which the interviewees gained information about these topics. Antimicrobial resistance is a great public health concern [23] and has become an important issue that needs to be addressed at all health sector levels. WHO urged all member countries to strongly promote measures for preventing antimicrobial resistance. There was a significantly higher proportion of healthcare providers with experience in RUD training among males (65.0%) than among females (35.3%). Since RUD trainings were usually held at a provincial level, far from the residency for most interviewees, women might have hesitated to participate. To change prescription practices, training opportunities should be increased [24].

More than 70% of interviewees had concerns about the quality of antibiotics. A survey on the quality of drugs conducted in 2012 revealed that the quality of drugs in Lao PDR, especially antimalarial drugs, was not well controlled [25]. Self-medication with antibiotics is also a great concern in Lao PDR. In our study, 85.2% of healthcare providers had concerns about self-medication. A study demonstrated that 55.5% of people with mild diseases in a peri-urban area were self-medicated at home [26].

Almost all interviewees (98.1%) stated that compliance with the guidelines would contribute to more appropriate antibiotic prescriptions. In addition, 61.1% of them strongly agreed that regulation is effective for RUD. Regulations for antibiotic prescription are important for improving prescription practices [27]. Policy makers should consider developing antibiotic prescription regulations.

Since there were only six healthcare providers at the PH, a comparison of responses between interviewees from the PH and the DHs did not seem meaningful. Another limitation of the survey among healthcare providers was that the subjects were the employees who voluntarily participated in this interview. In addition, the information was obtained through interviews rather than from objective records, which might have led to some inaccuracy.

### Actual antibiotic prescription based on medical records

Several factors may influence antibiotic prescription. Although data on this was not available in this study, requests from family members could be a strong contributor [28, 29]. Accurate diagnosis of viral or bacterial infection is also important for appropriate antibiotic use, but reliable diagnostic techniques are not widely available in Lao PDR. Patients were diagnosed based on their symptoms and limited laboratory tests. Without laboratory tests, it is difficult to rule out viral infections from URTI [30].

On average, 2.4 drug items were prescribed at the PH and 2.8 drug items were prescribed at the DHs. Although the average was slightly higher than the WHO reference value (2 items per visit), it was similar to values found in studies of the WHO African region (2.4 to 3.5 items) [31] and relatively higher than values noted in Ethiopia (1.9 items) [32]. Antibiotics were prescribed for 68.8% (106/154) of patients at the PH and 70.9% (299/422) at the DHs.

This study found that drugs on the EDL were frequently used (83.4%). The use of generic drugs was 80.7%, which is higher than the rate reported in a 2012 survey demonstrating that the majority of doctors (55.2%) prescribed generic antibiotics [29]. It was also found that 97.2% of antibiotic prescriptions at the PH and 97.7% at the DHs were first-line antibiotics. Beta-lactams were the most frequently prescribed for U5 (87.2%). The STG recommendation on first-line antibiotics was implemented in those hospitals. A similar frequency of first-line antibiotic use was observed in Bahrain [33], and amoxicillin was frequently used to treat URTIs in primary care settings [34]. The present study found inappropriate doses and periods of antibiotic prescription, and the right doses were prescribed significantly more often at the PH (52.8%) than at the DHs (24.4%). Thus, more attention is needed with respect to the prescription of appropriate antibiotic doses and prescription periods.

In many developing countries, antibiotic prescription for common cold is a public health concern [35–37]. Until recently, antibiotics were frequently used for common cold in Lao PDR. A study in 2004 demonstrated that antibiotics were prescribed for 41.0% of patients with non-pneumonia respiratory infections (*n* = 269) [38]. Prescription practices have been monitored by the government since 2013. At the beginning of the monitoring period, antibiotics were prescribed for almost 40% of common cold cases in public primary healthcare settings in Lao PDR. The health authority then urged practitioners to avoid such inappropriate practices. The present study revealed that antibiotics were prescribed for only 4.9% (8/164) of those with common cold. However, it was likely that U5 patients were given antibiotics before or after their visit to the health facilities, as antibiotics are sold in all private pharmacies without prescription.

There were several limitations to this study. First, the number of healthcare providers was limited, especially at the PH, as the study focused on the medical staff in OPDs. Second, the relationship between the interviewees’ responses to the questionnaire and their actual prescription practices was difficult to investigate. Third, the diagnosis was not confirmed by laboratory tests in all U5 patients, nor was the accuracy of the data recorded in the filing system in those health facilities. Finally, although this study was conducted in the largest province of Lao PDR, our sample may not represent the whole country.

In conclusion, the behaviors and prescription practices for U5 with common cold or URTI among healthcare providers at a PH and four DHs in Savannakhet Province were identified. They responded to follow the guideline for antibiotic prescription. While an appropriate number of generic first-line antibiotics from the EDL were prescribed, the prescribed doses and the periods of use were not appropriate. With a view to further improve prescription practices, regulations by the Lao PDR Ministry of Health are necessary to allow for better control of antibiotic prescriptions, which would be crucial in decreasing antimicrobial resistance and improving treatment outcomes.

## Abbreviations

AAW: Antibiotic Awareness Week
ADR: Adverse drug reaction
CI: Confidence interval
DH: District hospital
DTC: Drug and therapeutics committee
EDL: Essential drug list
INN: International non-proprietary name
Lao PDR: Lao People’s Democratic Republic
MoH: Ministry of Health
OPD: Outpatient division
OR: Odds ratio
PH: Provincial hospital
RUD: Rational use of drug
STG: Standard Treatment Guideline
U5: Children under 5 years of age
URTI: Upper respiratory tract infections
WHO: World Health Organization
